# Host life-history strategy is a critical determinant of virulent phage infection propensity

**DOI:** 10.1093/ismejo/wrag168

**Published:** 2026-07-13

**Authors:** Chuncheng Wu, Jacques Mathieu, Cory Schwarz, Madelyn Whitaker, Jenny A Laverde Gomez, Pedro J J Alvarez

**Affiliations:** Department of Civil and Environmental Engineering, Rice University, Houston, TX 77005, United States; Department of Civil and Environmental Engineering, Rice University, Houston, TX 77005, United States; Department of Civil and Environmental Engineering, Rice University, Houston, TX 77005, United States; Department of Chemical and Biomolecular Engineering, Rice University, Houston, TX 77005, United States; Department of Civil and Environmental Engineering, Rice University, Houston, TX 77005, United States; Department of Civil and Environmental Engineering, Rice University, Houston, TX 77005, United States; Department of Chemical and Biomolecular Engineering, Rice University, Houston, TX 77005, United States; Rice WaTER Institute, Rice University, Houston, TX 77005, United States

**Keywords:** bacteriophage, phage lifestyle, virulent phage, lysogeny, host life-history strategy, microbial genomics

## Abstract

Bacteriophages shape microbial communities through two major lifestyles: virulent (obligately lytic) and temperate (capable of lysogeny). Prevailing phage ecology frameworks focus on how environmental conditions, host density, and physiological state modulate infection modality. This perspective overlooks how host traits exert selective pressure on the distribution of virulent and temperate lifestyles across bacterial species, which limits understanding of phage ecology. To address this critical knowledge gap, we adopt a host-centric, trait-based perspective and use 5821 complete bacterial genomes to build a host life-history space predominantly defined by genome size, metabolic capacity, and growth rate potential. After mapping phage lifestyle association signals, prophage burden formed a continuous gradient across this space. Also, virulent phage association was positively correlated with prophage burden, revealing a nested structure of lifestyle signals. Functional trait analysis identified enrichment of resource-acquisition modules underlying both temperate and virulent associations. Overall, these findings indicate that phage lifestyle is significantly influenced by host life-history strategies, highlighting fast-growing, metabolically versatile hosts as favorable targets for virulent phage isolation and biocontrol applications.

## Introduction

Bacteriophages (or phages) are the most abundant biological entities on Earth [1], and their virulent (obligately lytic) and temperate (capable of lysogeny) lifestyles have distinct ecological consequences [2]. Virulent phages regulate microbial diversity and evenness [3, 4] through host lysis, imposing top-down control [3] and influencing global carbon flux [5, 6], whereas temperate phages integrate into host genomes as prophages [7, 8], conferring traits such as superinfection immunity [1, 9] and facilitating horizontal gene transfer [10, 11] that shapes bacterial ecology and evolution. Given their ecological importance, discerning the factors that determine virulent or temperate phage lifestyles across bacterial hosts is critical for understanding ecosystem dynamics and guiding phage-based applications.

The determinants of phage lifestyles are multifaceted and complex, largely stemming from phage reliance on host-derived resources for proliferation, including nucleotide and amino acid precursors and the host translational machinery [12, 13]. Although environmental conditions and microbial population dynamics influence the immediate availability of these resources during infection, intrinsic physiological and metabolic traits of the host define baseline differences in resource acquisition [14, 15]. It remains unclear whether such intrinsic host traits systematically shape the distribution of phage lifestyle associations across species.

Phage ecology has traditionally emphasized environmental and density-driven control of dominance of lytic versus lysogenic strategies at the community level, exemplified by frameworks such as Kill-the-Winner (KtW), Piggyback-the-Winner (PtW), and Piggyback-the-Loser (PtL) [16–18]. KtW predicts strong lytic control of dominant, fast-growing hosts, whereas PtW suggests lysogeny can be favored under high host density. In contrast, PtL proposes that lysogeny prevails when host growth is limited or conditions are unfavorable for lytic infection [16–18]. Complementary experimental studies have shown that host physiological state [14, 15, 19], such as host growth rate [14, 15], energy status [20, 21], and stress responses [22, 23], can strongly affect phage infection outcomes, including burst size, latent period, replication efficiency, and lysis–lysogeny decisions, within individual host–phage systems [14, 15, 19]. Yet, none of these frameworks explicitly integrates host-intrinsic traits as a key dimension of phage lifestyle associations.

Environmentally centered ecological frameworks treat hosts inhabiting the same environment as functionally equivalent units, which they are not, overlooking genetic differences among host species. Likewise, physiological studies focus primarily on model host-phage systems (e.g. T7, T4, and λ) and examine short-term infection dynamics, rather than providing a predictive understanding of stable phage lifestyle associations across different bacterial species. In reality, even under identical environmental conditions, disparate bacterial species differ in energetic efficiency [24, 25], carbon-use strategies [26, 27], SOS regulatory dynamics [28, 29], and other host-intrinsic traits [30, 31]. Existing studies of phage lifestyle associations largely emphasize phylogenetic patterns and rarely integrate host functional traits [32, 33]. Thus, a fundamental question remains unanswered: ‘do host-intrinsic traits systematically influence phage lifestyle propensity?’ Addressing this question will refine our understanding of host-associated constraints shaping phage lifestyles and enhance the development of phage-based applications.

Here, we take a host-centric, trait-based perspective to investigate how bacterial life-history strategies relate to phage lifestyle distributions across broad phylogenetic scales. By integrating large-scale prophage detection, virulent phage-host associations, and quantitative host trait profiles across databases with thousands of bacterial genomes, we systematically examine how host intrinsic properties bias phage lifestyle patterns. Motivated by efforts to identify host backgrounds favorable for isolating virulent phages to enhance phage-based biocontrol, we show that phage lifestyle associations are non-randomly distributed across bacterial species. Hosts associated with virulent phages occur within prophage-rich clades and exhibit traits linked to resource acquisition and rapid growth. This framework provides an integrative view of how host traits shape phage–host interactions and reveals how phage lifestyle selection may guide virulent phage isolation and the development of phage-based applications.

## Materials and methods

### Genome dataset and taxonomy unification

We adopted a conservative genome-selection strategy and retained only assemblies classified as “Complete Genome” in the NCBI RefSeq database (October 2024). A total of 5942 bacterial genomes met this criterion, whereas assemblies at the chromosome, scaffold, or contig level were excluded. Genome quality was subsequently assessed using CheckM and Genome Taxonomy Database (GTDB, bac120 r226) [34], and only genomes with completeness ≥50%, contamination ≤10%, and a quality score (completeness −5 × contamination) >50 were retained [35]. Genomes failing these criteria or exhibiting evident assembly anomalies were excluded, resulting in a final dataset of 5821 high-quality genomes. Of these, 5803 (99.69%) were isolate genomes, 14 (0.24%) were metagenome-assembled genomes (MAGs), and 4 (0.07%) had unknown origin. No additional inclusion or exclusion criteria were applied. The retained genomes represented 5457 species according to the GTDB (bac120 r226).

Because bacterial functional traits reflect long-term species-level strategies whereas certain genome features (e.g. CRISPR–Cas systems) vary within species, analyses were conducted at different resolutions. Multitable co-inertia analysis (MCOA), a multitable ordination approach that integrates multiple feature datasets into a shared low-dimensional space [36], and microTrait, a genome-based framework for inferring microbial ecological traits from genomic content [37], were performed at the GTDB-defined species level by aggregating genomes within each species (mean values), whereas other analyses were primarily conducted at the genome level.

### Construction of host life-history strategy space by MCOA

To define a host life-history strategy space, MCOA was performed [36]. Unlike ordination methods based on a single distance matrix (e.g. PCoA or NMDS), MCOA integrates multiple feature tables and identifies axes that maximize shared covariance across them. This allows us to capture consensus structure across independent representations of host biology rather than patterns driven by any single dataset. Genomes belonging to the same GTDB species were aggregated by mean values to generate four species × feature matrices: (i) KEGG pathway proportion, (ii) COG functional category proportion, (iii) CAZy family proportion, and (iv) genomic traits (GC content, genome length, 16S rRNA operon copy number, anti-phage defense gene counts, and predicted doubling time).

KEGG pathway composition and COG functional composition were derived from eggNOG-mapper v2.1.13 [38], using eggNOG DB v5.0.2 [39]. Carbohydrate-Active enZYmes (CAZy) family [40] composition was obtained using dbCAN v2.0.11 [41]. 16S rRNA operon copy number was predicted using barrnap v0.9 [42], and anti-phage defense genes were identified using DefenseFinder [43]. The predicted doubling time was estimated by gRodon2 [31]. When using gRodon2, Weissman *et al.* [31] proposed a threshold of 5 h minimal doubling time to distinguish copiotrophic and oligotrophic growth regimes; therefore, species with predicted doubling times exceeding this threshold were considered to belong to the slow-growth (oligotrophic) regime.

All functional features were expressed as relative abundance proportions, calculated as the number of proteins assigned to a given functional category divided by the total number of annotated proteins in that genome. Functional features present in fewer than 10% of genomes across the dataset were removed prior to analysis. For genomic traits, ln(x + 1) transformation was applied except for GC content.

MCOA was performed in R using the ade4 package [44]. Principal component analysis was first conducted for each table, retaining the first three components, followed by co-inertia analysis to generate shared species coordinates (SynVar), which represent the consensus positions of species along the synthetic axes maximizing covariance across all input tables. The first two axes (MCOA1 and MCOA2) were used for downstream analyses. To interpret MCOA1 and MCOA2, key contributing variables were identified across all input features by retaining variables that met all of the following criteria: linear model *P* < .001, *R*^2^ > 0.20, and ranked within the top 50 contributors to the corresponding axis.

High and low ends of the MCOA1 and MCOA2 axes were defined as the mean values of genomes within the top and bottom 10% along each axis.

### CRISPR–Cas defense profiling

CRISPR–Cas systems were profiled using CRISPRCasTyper (cctyper v1.8.0) [45]. For each genome, we summarized CRISPR–Cas system integrity status and CRISPR spacer burden (spacer count), which were used in downstream analyses.

### Prophage identification and quality filtering

Candidate prophage regions were predicted using PhiSpy v4.2.21 [46], a machine-learning-based framework that integrates multiple compositional and similarity-derived features for prophage identification. To reduce potential false-positive predictions arising from genomic islands and other mobile genetic elements with prophage-like characteristics, candidate regions predicted by PhiSpy were subjected to an additional filtering procedure. Genes within candidate regions were predicted using Prodigal v2.6.3 [47] and annotated using HMMER v3.4 [48] against merged PHROG (Prokaryotic Virus Remote Homologous Groups) [49] and VOG (Virus Orthologous Groups) [50] databases. Insertion sequences (IS) were detected using ISEScan v1.7.3 [51]. Regions were retained as high-confidence prophages if they contained at least one integrase, at least one structural protein marker, and had IS content <25%. IS content was defined as the fraction of nucleotides annotated as IS-related sequences relative to the total region length. Regions failing criteria were excluded. This conservative filtering strategy was intended to enrich bona fide prophage regions while minimizing the inclusion of highly degraded mobile genetic elements.

### Identification of virulent phage hosts and association with host genomic traits

Candidate virulent phage genomes were collected from the NCBI Virus database and PhageScope [52]. Only complete phage genomes were retained. Phage–host matching was performed using strict species-level host name matching: host names recorded in phage metadata were first matched to host genome names downloaded from NCBI, and then mapped to GTDB species, yielding species-level phage–host associations.

To infer phage lifestyle, phage genomes were gene-called using Prodigal v2.6.3 and annotated using HMMER against merged PHROG and VOG databases. At the genome level, we assessed the presence of integrase, terminase large subunit (terL), and lysis-associated genes (e.g. holin, endolysin, spanin). Lifestyle was assigned as follows: phages with integrase were classified as temperate; phages lacking integrase but containing both terL and lysis genes were classified as strong virulent; phages lacking integrase and containing terL but lacking lysis genes were classified as virulent; and phages lacking these hallmark genes were classified as uncertain. Using this framework, 12 807 phages (80.2%) were classified as temperate, 2150 (13.5%) as strong virulent, 21 (0.1%) as virulent, and 982 (6.2%) as uncertain. Only strong virulent phages were used to define virulent phage hosts in this study, providing a conservative lifestyle assignment to reduce potential misclassification.

At the host genome level, we tested associations between virulent phage presence (virulent count >0) and host lysogeny status (lysogen vs non-lysogen), as well as intact CRISPR–Cas presence (intact vs non-intact), using 2 × 2 contingency tables with Fisher’s exact test or Pearson’s χ^2^ test as appropriate, reporting odds ratios (OR). Genome size differences between virulent phage hosts and species without virulent phage associations were assessed using the Wilcoxon rank-sum test. Spacer burden was compared only within the subset of genomes encoding intact CRISPR–Cas systems, to avoid zero inflation caused by system absence, using the Wilcoxon rank-sum test.

To evaluate whether prophage burden significantly explained the probability of detecting virulent phage signals, we modeled virulent presence as a binary variable at the GTDB species level using binomial logistic regression. Prophage count was modeled using natural splines (df = 4) to allow non-linear relationships. Model significance was assessed by likelihood ratio tests (LRT) comparing the spline model to an intercept-only null model, and model fit was further compared using AIC.

### Spatial distributions of phage lifestyle-associated signals in MCOA space

To evaluate whether phage lifestyle-associated signals were non-randomly distributed across host life-history strategy space, we performed complementary quadrant-level, axis-level, and centroid-based analyses. For quadrant-level analyses, the MCOA space was divided into four quadrants using the zero values of MCOA1 and MCOA2 as boundaries. Axis-level analyses evaluated enrichment along individual MCOA axes by comparing the proportions of genomes located on either side of an axis. Centroid-based analyses compared the mean positions of groups within the MCOA space to quantify overall shifts in their distributions. Enrichment of virulent phage hosts in Quadrant I (MCOA1 > 0, MCOA2 > 0) was assessed using a 2 × 2 contingency table (virulent phage hosts/species without virulent phage associations × in-Q1/not-in-Q1) and Fisher’s exact test. For lysogens, enrichment along individual axes was assessed by comparing the proportion of lysogens and non-lysogens in the positive region of each axis (e.g. MCOA1 > 0) using 2 × 2 contingency tables and χ^2^ tests.

To evaluate continuous spatial shifts without relying on discrete region classification, distributions of phage-associated hosts and their corresponding comparison groups along MCOA1 and MCOA2 were compared using the Wilcoxon rank-sum test. Group centroid coordinates were calculated as the mean MCOA1 and MCOA2 values for each host category to quantify directional displacement within the strategy space.

Significance of centroid displacement for virulent phage hosts was assessed using permutation tests (10 000 iterations) based on Euclidean distance.

### microTrait analysis of host functional investment and its association with prophage burden and virulent phage presence

Host functional traits were inferred using microTrait [37], a genome-based trait prediction framework that identifies ecologically relevant microbial traits from genomic features using hidden Markov model-based gene detection and rule-based trait assignment [37]. microTrait generates trait profiles describing microbial resource acquisition, resource use, stress tolerance, and life-history strategies from genome sequences. Trait values generated by microTrait were aggregated at the GTDB species level and used for downstream analyses.

At the GTDB species level, microTrait associations with phage lifestyle signals were evaluated using two complementary analyses. First, microTrait distributions were compared between virulent phage hosts and species without virulent phage associations. Effect sizes were quantified using Cliff’s delta, a non-parametric measure of distributional separation defined as the probability that a randomly selected observation from one group exceeds a randomly selected observation from the other group minus the reverse probability [53]. Values range from −1 to 1, with 0 indicating complete overlap between groups. Following the effect-size interpretation proposed by Romano *et al.* [54], values below 0.147 are generally negligible. We therefore retained only traits with *q* ≤ 0.01 and |Cliff’s delta| ≥ 0.20 to focus on biologically meaningful group differences. Second, monotonic associations between microTrait traits and prophage burden were quantified using Spearman’s rank correlation coefficient (ρ), with traits retained at *q* ≤ 0.01 and |ρ| ≥ 0.20.

To identify convergent host functional signatures, we retained traits that were significantly enriched in virulent phage hosts and positively correlated with prophage burden (both *q* ≤ 0.01; Cliff’s delta ≥0.20; Spearman’s ρ ≥ 0.20).

### Assessment of MCOA robustness against taxonomic and growth-strategy sampling bias

We performed a stratified bootstrap subsampling analysis to assess the robustness of the MCOA-derived host life-history strategy space and associated phage patterns against taxonomic bias and uneven representation of host growth strategies. In each iteration, species were first stratified into fast-growing (predicted doubling time < 5 h) and slow-growing (≥ 5 h) groups, using the threshold proposed by Weissman *et al.* [31] to distinguish copiotrophic and oligotrophic growth regimes in gRodon2-based growth predictions, from which 500 species were sampled from each group using a phylum-balanced procedure. MCOA was then recomputed for each dataset. The stability of MCOA space, genome positions, and downstream spatial patterns was evaluated across iterations. Details are provided in Supplementary Information.

### Statistical analyses

All statistical tests were two-sided. Raw *P* values were calculated for all hypothesis tests and are reported as appropriate. For analyses involving single comparisons, *P* < .05 was considered statistically significant unless otherwise specified.

Categorical associations were assessed using Fisher’s exact test or Pearson’s χ^2^ test, with effect sizes reported as OR. Continuous variables were compared using the Wilcoxon rank-sum test, and effect sizes were quantified using Cliff’s delta. Monotonic associations were evaluated using Spearman’s rank correlation coefficient (ρ). Regression-based analyses and permutation procedures are described in the corresponding sections above.

When multiple hypotheses were tested simultaneously, raw *P*-values were adjusted using the Benjamini–Hochberg false discovery rate (FDR) procedure. Adjusted *q* values ≤0.01 were considered statistically significant unless otherwise specified.

Effect-size thresholds (|Cliff’s delta| ≥ 0.20; |Spearman’s ρ| ≥ 0.20) were applied in trait-level analyses to focus on biologically meaningful associations.

### Computational resources

All analyses were performed on the Rice University NOTSX high-performance computing cluster. All scripts used for data processing are publicly available at GitHub (https://github.com/ccw-rice/phage-host-trait-data).

## Results

### Genome size, resource acquisition, growth rate, and energy use define the host life-history strategy space

**Figure 1:**
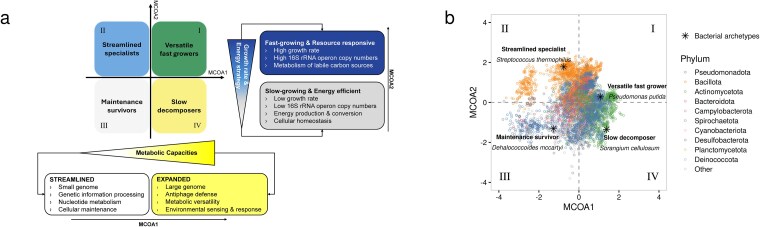
Host life-history space defined by MCOA identifies four quadrants corresponding to heuristic bacterial archetypes. (a) MCOA1 captures variation in genome size and metabolic capacity, whereas MCOA2 reflects growth rate and energy-allocation strategies. We partitioned the two-dimensional MCOA space into four quadrants with distinct life-history characteristics. Quadrant I comprises bacteria with larger genomes and broader metabolic capacity, coupled with higher potential growth rates. These taxa can degrade and utilize complex organic substrates, rapidly acquire labile carbon sources to support fast growth, and exhibit enhanced environmental responsiveness and stress tolerance, representing an expanded-metabolism strategy that enables rapid biomass accumulation when resources become favorable (e.g. *Pseudomonas putida*). Quadrant II is dominated by bacteria with smaller, streamlined genomes, relatively specialized metabolic repertoires, and high growth potential. These taxa exhibit narrow and well-defined metabolic profiles and efficiently channeled limited cellular energy into rapid biomass accumulation, representing a specialized fast-growing strategy (e.g. *Streptococcus thermophilus*). Quadrant III comprises bacteria with small genomes and low growth rates, with cellular energy and resources primarily invested in stress tolerance, energetic efficiency, and homeostatic maintenance, consistent with a conservative strategy centered on long-term survival and stability (e.g. *Dehalococcoides mccartyi*). Quadrant IV includes bacteria with large genomes and high metabolic potential for complex organic matter utilization but relatively low growth rates. These taxa preferentially allocate cellular energy toward efficient energy production and homeostasis maintenance rather than rapid biomass accumulation, consistent with a maintenance-oriented strategy with high metabolic capability but slower growth (e.g. *Sorangium cellulosum*). (b) Distribution of all genomes in the MCOA-defined space. Representative microbial examples are highlighted to illustrate how well-characterized taxa map onto distinct regions of the strategy space.

**Figure 2:**
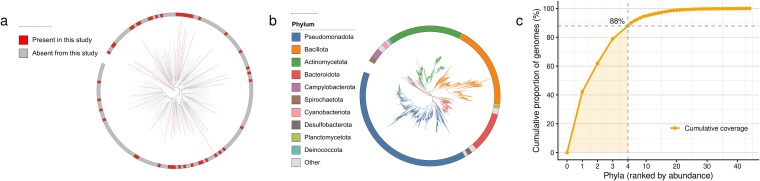
Phylogenetic distribution of the considered genomes reveals uneven representation of phyla. (a) Phylum-level phylogenetic coverage of the considered genomes mapped onto the GTDB reference tree (bac120 r226). The dataset spans 44 of the 183 phyla currently defined in GTDB. (b) Phylogenetic distribution of the considered genomes. (c) Cumulative proportion of genomes represented by phyla ranked from most to least abundant. The curve illustrates the taxonomic imbalance of the dataset, with the four most abundant phyla accounting for 88% of all genomes.

To move beyond frameworks centered on environmental conditions, host density, and host physiological state, we applied MCOA to integrate species-aggregated features, resulting in a host life-history strategy space (Fig. 1). This analysis was based on 5821 high-quality complete bacterial reference genomes standardized using the GTDB framework [34]. These genomes span 44 of the 183 phyla currently defined in GTDB (Fig. 2). However, taxonomic representation was uneven, with the four most abundant phyla accounting for 88% of all genomes (Fig. 2c); this reflects known biases in reference genome collections [55, 56]. The first two axes of MCOA captured the major sources of variation, explaining 34% (MCOA1) and 18% (MCOA2) of total host trait variance (Fig. 1).

Genome size contributed most strongly to MCOA1 (*P* < .01, *R*^2^ = 0.87; Fig. S1). The low end of MCOA1 was characterized by smaller genomes (mean of bottom decile = 1.3 Mb) comprised primarily of core cellular functions, representing a streamlined strategy prioritizing essential processes with reduced proportional investment in more complex metabolism. In contrast, the high end corresponded to larger genomes (mean of top decile = 8.0 Mb) with higher GC content (mean of top decile = 69%, mean of bottom decile = 33%) and greater metabolic versatility, broader resource-acquisition capacity, and enriched environmental sensing, regulatory and defense functions, consistent with adaptation to more complex environments [57]. Thus, MCOA1 captured a continuous gradient characterized by genome size and metabolic capacity.

MCOA2 primarily reflected variation in host growth rate and energy allocation. Generation time was the dominant contributor and was negatively associated with MCOA2 (*P* < .01, *R*^2^ = 0.55; Fig. S2). The low end was enriched in energy production and maintenance-related functions, indicating strategies favoring efficient energy utilization and cellular homeostasis rather than rapid growth. The high end was associated with faster growth rate (reflected by doubling times, top decile mean = 0.9 h, mean of bottom decile = 9.1 h), greater growth potential (reflected by 16S rRNA operon copy numbers, top decile mean = 8.0, mean of bottom decile = 1.5), and enrichment in labile carbon uptake and metabolism pathways. These features indicate a strategy centered on fast environmental responsiveness and rapid growth, with a preference for readily degradable carbon substrates. Accordingly, MCOA2 described a trade-off between rapid biomass accumulation and cellular homeostasis maintenance.

Based on these ecological interpretations of MCOA1 as a gradient of genome size and metabolic capacity and MCOA2 as a trade-off between rapid biomass accumulation and cellular homeostasis maintenance, we divided the two-dimensional strategy space into four quadrants defined by MCOA1 = 0 and MCOA2 = 0, representing distinct combinations of metabolic capacity and growth investment (Fig. 1a): versatile fast growers (Quadrant I), streamlined specialists (Quadrant II), maintenance survivors (Quadrant III), and slow decomposers (Quadrant IV).

### Virulent and temperate phage associations are concentrated in metabolically versatile, fast-growing hosts

To examine how bacterial life-history strategies may relate to phage lifestyles, we established two independent pipelines (Figs S3 and S4) to detect prophages within each bacterial genome and identify virulent phages.

#### Lysogeny is widespread and associated with genome expansion and elevated defense signatures

Across the 5821 genomes considered, 78% contained at least one prophage. Overall, 49% of genomes were poly-lysogens, with one species (*Sodalis glossinidius*) harboring up to 24 prophages (Fig. S5), indicating widespread lysogeny across bacterial species. Lysogen genomes were often significantly larger than non-lysogens (mean genome size = 4.4 Mb versus 2.9 Mb, median genome size = 4.1 Mb versus 2.7 Mb), and this size difference persisted even after removing detected prophage sequences (mean genome size = 4.3 Mb versus 2.9 Mb, median genome size = 4.0 Mb versus 2.7 Mb), suggesting that genome expansion in lysogens cannot be explained solely by prophage contributions. Prophage number generally increased with host genome size, whereas prophage density peaked in 1–2 Mb genomes and declined in larger genomes (Fig. S6).

With respect to host defense-associated features, lysogens showed a significantly higher proportion of genomes encoding complete CRISPR–Cas systems than non-lysogens (χ^2^ test, *P* = 2.1 × 10^−12^). They also exhibited significantly higher CRISPR spacer counts (Wilcoxon rank-sum test, *P* = 3.8 × 10^−9^). Moreover, genomes carrying CRISPR–Cas systems had slightly higher prophage numbers (Wilcoxon rank-sum test, *P* = 8.5 × 10^−13^), and prophage number was positively correlated with spacer count (Spearman ρ = 0.08, *P* = 5.2 × 10^−9^). Together, these results indicate that lysogens display systematic differences in genome size and defense-related genomic signatures consistent with long-term host–phage interactions.

#### Prophage burden varies continuously with resource acquisition and growth potential across host life-history strategy space

**Figure 3:**
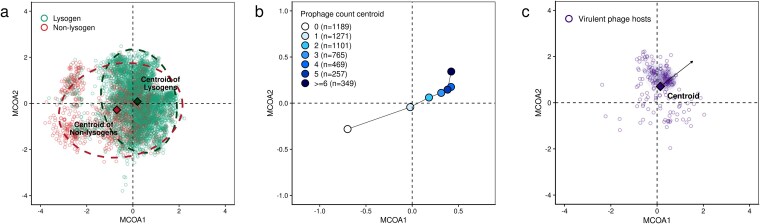
Prophage burden forms a continuous gradient in host life-history strategy space, with virulent phage hosts enriched in the same region of Quadrant I. (a) Distribution of lysogen and non-lysogen genomes mapped onto the MCOA-defined host life-history strategy space. Centroids indicate the mean position of each group, and dashed ellipses represent the 95% confidence regions of the corresponding distributions. (b) Centroid positions of genomes grouped by prophage count, showing a continuous trajectory across the MCOA plane. Numbers in parentheses indicate the number of genomes per group. (c) Distribution of virulent phage host genomes in the same space. Points represent genomes associated with at least one virulent phage, and the filled diamond indicates the group centroid. The shaded region represents a two-dimensional kernel density estimate of virulent phage host distribution. The arrow indicates the direction of significant enrichment toward Quadrant I. Virulent phage hosts overlap with prophage-rich regions and exhibit a distribution shift primarily along MCOA2.

To determine how lysogeny-associated signals are distributed within the host life-history strategy space, we mapped genomes classified by prophage presence (lysogen versus non-lysogen) onto this space (Fig. 3a). Both groups showed extensive overlap, though lysogens were biased toward positive MCOA1 values, whereas non-lysogens covered the negative MCOA1 region more broadly and evenly, as reflected by a higher centroid along MCOA1 for lysogens compared to non-lysogens (0.20 versus −0.70; Wilcoxon rank-sum test, *P* < .01) and a greater proportion of lysogens occupying the positive MCOA1 region (64% versus 37%; χ^2^ test, *P* < .01).

To further resolve how prophage burden is distributed in the host life-strategy space, we grouped genomes by prophage number and computed centroid coordinates for each group. We observed a consistent and approximately monotonic displacement of group centroids with increasing prophage burden, shifting from negative toward positive MCOA1 values and upward along MCOA2 (Fig. 3b), without forming discrete clusters. When prophage burden reached ≥2, centroid positions stabilized within Quadrant I (defined as MCOA1 > 0 and MCOA2 > 0), corresponding to host strategies characterized by larger genomes, expanded metabolic capacity and stronger resource responsiveness.

#### Virulent phage hosts are nested within prophage-rich host backgrounds and become more prevalent with increasing prophage burden

We examined host features and MCOA distributions of virulent phage hosts, defined as species associated with at least one virulent phage genome, and species without virulent phage associations. Under the GTDB taxonomy framework, 171 of 5457 bacterial species (3%) showed association with virulent phage genomes. Compared with hosts without virulent phage associations, virulent phage hosts were modestly enriched among lysogens (χ^2^ test, *P* = .024), indicating that virulent phage signals are more frequently detected in lysogenic backgrounds. Consistent with this enrichment, the genomes of virulent phage hosts were slightly larger on average than those of species without virulent phage associations (4.2 Mb versus 4.1 Mb; Wilcoxon rank-sum test, *P* = .045), although the magnitude of this difference was limited.

The prevalence of complete CRISPR–Cas systems did not differ significantly between the two groups (virulent phage hosts vs. species without virulent phage associations, χ^2^ test, *P* = .101). This lack of association persisted after stratifying by lysogen status, with no significant difference in CRISPR prevalence among either lysogen (χ^2^ test, *P* = .122) or non-lysogen hosts (χ^2^ test, *P* = .969). Among host genomes encoding complete CRISPR–Cas systems, virulent phage hosts exhibited significantly fewer CRISPR spacers than species without virulent phage associations (means 36.6 versus 62.5; medians 28 versus 42; Wilcoxon rank-sum test, *P* = .002), although the magnitude of this reduction was modest (Cliff’s δ = −0.21).

Mapping virulent phage hosts onto the MCOA space revealed significant enrichment within Quadrant I (Fisher’s exact test, OR = 1.85, *P* = 1.5 × 10^−4^; Fig. 3c). This enrichment was primarily driven by a pronounced positive shift along MCOA2 (Wilcoxon rank-sum test, *P* < 2.2 × 10^−16^), with no overall shift along MCOA1 (*P* = .500). Permutation tests further confirmed that the centroid of virulent phage host genomes was significantly displaced toward Quadrant I (*P* < 1 × 10^−4^). Collectively, virulent phage hosts occupied positions that substantially overlapped with prophage-rich lysogens, indicating convergence on similar host life-history strategy endpoints.

**Figure 4:**
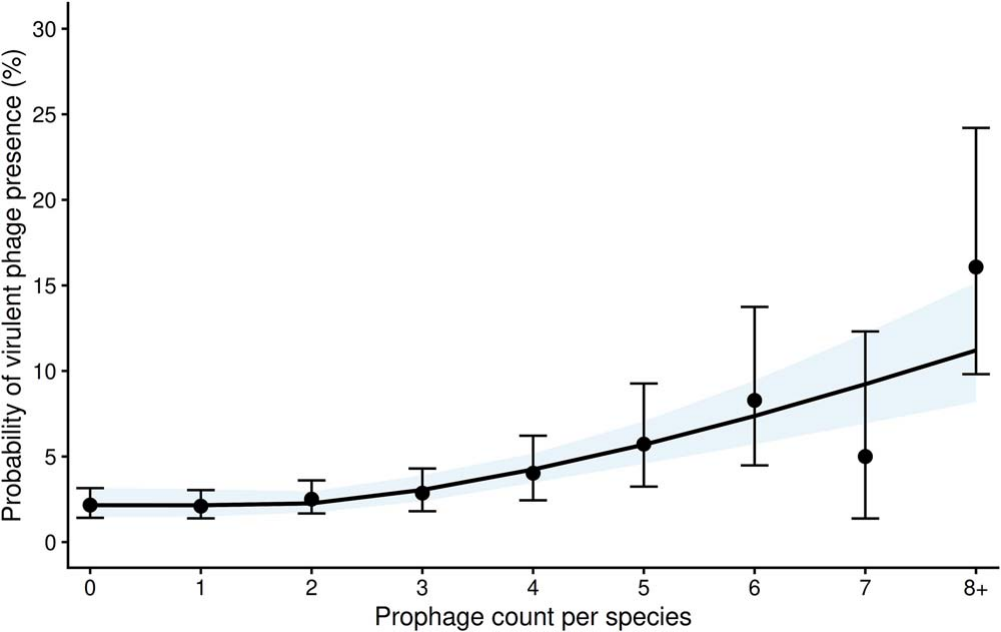
Virulent phage detection probability increases with prophage burden. Virulent phage presence was modeled as a binary trait at the GTDB species level (has virulent = 1 if virulent count >0, otherwise 0) using binomial logistic regression. Prophage burden (prophage count) was fitted using natural splines (df = 4). Points represent the observed proportion of virulent phage hosts (mean ± 95% confidence intervals) within each prophage-count category. The solid line shows the fitted probability from the spline logistic model, and the shaded band indicates the 95% confidence interval of the fitted relationship.

To determine whether virulent phage signals show a tighter statistical association with prophage burden, we quantified, at the species level, the probability of detecting virulent phages under different prophage burden conditions. Virulent phage occurrence increased approximately monotonically with prophage burden (Fig. 4), remaining low at 0–2 prophages but increasing in higher-burden categories. This trend further provides evidence for convergent host associations of temperate and virulent phages in host life-history strategy space.

### Species lacking detectable phage signals are concentrated in slow-growing, maintenance-oriented hosts

A substantial subset of species lacked both detectable prophages and virulent phage associations (1164 of 5457 GTDB species; 21%). Rather than being randomly distributed across the host life-history strategy space, these species were significantly enriched in Quadrant III and depleted in Quadrants I and IV (Fig. S7, χ^2^ test, *P* < .001). This pattern indicates that the absence of detectable phage signals is associated with host strategies characterized by slower growth, smaller genomes, and greater investment in cellular maintenance.

### Robustness of host life-history strategy space and phage-associated patterns against taxonomic and growth-strategy sampling bias

To address both the strong taxonomic skew of the dataset and the potential overrepresentation of fast-growing taxa in reference genome collections, we performed a stratified bootstrap subsampling analysis. In each iteration, species were first stratified into fast-growing (predicted doubling time < 5 h) and slow-growing (≥ 5 h) groups based on gRodon2 predictions. Within each growth-strategy group, species were then sampled using a phylum-balanced procedure before recomputing the MCOA space. Across 50 bootstrap iterations, the major variables contributing to the MCOA axes remained highly consistent, and genome positions showed strong concordance with the full dataset. The continuous gradient of prophage burden and the spatial distribution of virulent phage host genomes were preserved across subsampled datasets (Figs S8 and S9), indicating that both the host life-history strategy space and associated phage patterns are robust against taxonomic sampling bias (see Supplementary for full results).

### Temperate and virulent phage associations converge on hosts with elevated resource acquisition potential

MCOA indicated that virulent phage hosts overlapped extensively with lysogens in host life-history strategy space and did not form a distinct partition (Fig. 3). Furthermore, genomes grouped by prophage numbers exhibited a clear continuous displacement along the MCOA axes (Fig. 3b). As the MCOA alone does not identify the specific functional dimensions shaping this trend, we applied microTrait [37] to quantify host genomic investment in ecological functions to discern a possible relationship between host traits and prophage burden.

**Figure 5:**
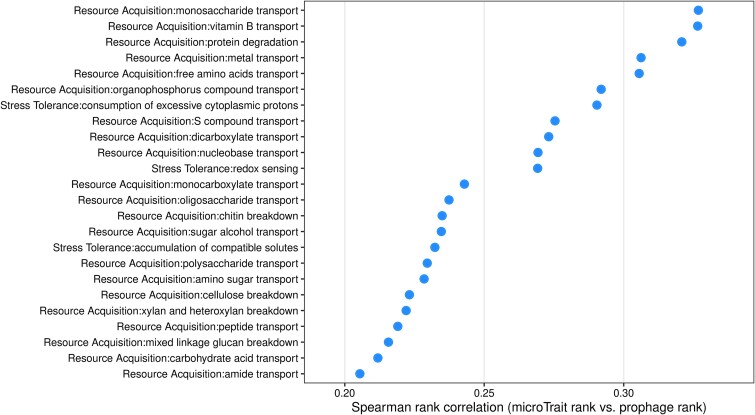
Prophage accumulation is positively associated with resource acquisition traits, including diverse nutrient transport and complex polysaccharide degradation. Monotonic associations between microTrait traits and prophage burden were quantified using Spearman’s rank correlation (−1 < ρ < 1) at the GTDB species level, followed by false discovery rate (FDR) correction. Each point represents one microTrait category, with the *x*-axis indicating Spearman’s ρ between microTrait rank and prophage rank. Traits with *q* ≤ 0.01 and |ρ| ≥ 0.20 were retained and visualized.

Prophage rank was consistently positively correlated with multiple resource-acquisition traits (Fig. 5). The strongest signals involved diverse transport functions (monosaccharide, free amino acid, vitamin B, organophosphorus compound, and organic acid transport) and complex carbon degradation pathways (cellulose, xylan/hemicellulose, and chitin), suggesting that prophage-rich hosts exhibit greater genomic investment in accessing diverse environmental resources. Together, these relationships provide a functional interpretation for the continuous host strategy spectrum revealed by MCOA. To assess whether these associations were driven by prophage-encoded cargo functions, we repeated the microTrait analysis after removing all predicted prophage regions from host genomes. Correlations remained highly consistent with the original analysis, indicating that the enrichment of resource-acquisition traits is not solely attributable to prophage sequence content (Fig. S10).

**Figure 6:**
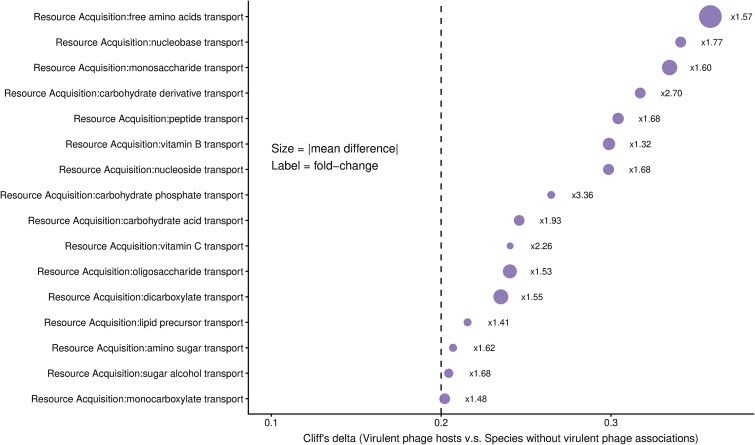
Virulent phage hosts exhibit broadly elevated resource acquisition traits, particularly diverse nutrient transport capacities. microTrait count traits were compared between virulent phage hosts and species without virulent phage associations at the GTDB species level. Effect sizes were quantified using Cliff’s delta (virulent phage hosts minus species without virulent phage associations), with statistical significance assessed by Mann–Whitney *U* tests followed by false discovery rate (FDR) correction. Traits with *q* ≤ 0.01 and |Cliff’s delta| ≥ 0.20 were retained. Points show Cliff’s delta for each trait, a non-parametric effect size ranging from −1 to 1 that quantifies the degree of separation between two distributions, where positive values indicate enrichment in virulent phage hosts and negative values indicate enrichment in species without virulent phage associations. Larger point sizes indicate larger absolute mean differences between groups, and text labels denote fold change (virulent phage hosts/species without virulent phage associations).

Given the monotonic association between prophage burden and resource acquisition traits, we further tested whether virulent phage hosts correspond to a distinct functional strategy space (Fig. 6). microTrait-based group comparisons showed that, relative to species without virulent phage associations, virulent phage hosts similarly exhibited consistent increases in resource acquisition traits (monosaccharide; oligosaccharide and carbohydrate-derivative; peptide and free amino acid; nucleoside/nucleobase; and vitamin transport), indicating that virulent phage hosts show broadly enhanced substrate uptake capacity rather than specialization for a single resource type.

To identify host functional traits jointly associated with virulent phage presence and prophage burden, we retained traits significantly enriched in virulent phage hosts and positively correlated with prophage burden, requiring significance and concordant directions in both tests (two-sided tests with *q* ≤ 0.01, |Cliff’s delta| ≥ 0.20, and Spearman’s ρ ≥ 0.20; Table S1). A total of 11 traits were shared between the virulent phage and prophage analyses, representing 69% (11/16) of virulent-associated traits and 46% (11/24) of prophage-associated traits. Notably, all shared traits (11/11; 100%) corresponded to resource acquisition modules (carbon transport, amino acid and peptide transport, nucleoside/nucleobase transport, vitamin transport, and carboxylate transport). Collectively, these signals indicate strong convergence between temperate and virulent phage associations in host functional strategies, centered on elevated broad-spectrum resource acquisition potential.

## Discussion

Classical phage ecology theories emphasize environmental, host-density, and physiological controls on phage infection dynamics, implicitly treating hosts as ecologically homogeneous within environments and overlooking differences in host characteristics. Whereas these frameworks explain short-term infection dynamics, they overlook whether intrinsic host traits shape the distribution of virulent versus temperate associations across species. This limits basic understanding of phage ecology and predictive power for phage-based applications. In developing a host-trait defined life-history strategy space and mapping virulent and temperate phage hosts within this matrix, we reveal that virulent phage hosts are strongly defined by host metabolic capacity and growth investment.

The host strategy axes capture key host differences relevant to phage interactions. Toward the positive end of MCOA1, hosts are more likely to maintain metabolic and biosynthetic states that support phage replication across diverse ecological contexts [58, 59], which increases the probability that a host constitutes a “replication-feasible” background, thereby facilitating the accumulation of phage-associated signals over evolutionary timescales. Because lytic replication requires rapid amplification within a limited time window, virulent phages are particularly sensitive to host-accessible energy flux and translational throughput [60]. Thus, the growth-investment state represented by MCOA2 may more directly constrain where virulent phage-associated signals become enriched.

Prophage burden did not partition hosts into discrete categories in MCOA space but instead formed a smooth and monotonic gradient along the host strategy axes. This suggests that lysogeny-associated signals reflect a cumulative ecological outcome along a host strategy continuum, rather than a binary preference confined to a fixed host type. Because prophage accumulation depends on both acquisition and retention probabilities, even subtle differences in these probabilities could, over long timescales, accumulate into a pronounced burden gradient. Continuous differences in host resource acquisition capacity and energy allocation may alter the likelihood of successful infection and integration [61], whereas the net fitness effects of prophages can shift with environmental conditions and selective pressures: when costs outweigh benefits, prophages may be purged, whereas under superinfection exclusion or adaptive advantages, they are more likely to be maintained [62, 63].

Virulent phage hosts did not form an independent cluster opposing prophage-rich hosts in the strategy space; instead, they overlapped extensively. Both signals share similar backgrounds along MCOA1, whereas the enrichment of virulent phage host is expressed as a systematic shift along MCOA2, toward stronger growth investment and rapid resource responsiveness. Thus, virulent phage hosts constitute a strategic subset nested within prophage-rich host backgrounds, rather than a fundamentally distinct type. This nested configuration echoes theoretical models of virulent–temperate coexistence and environmental surveys reporting the concurrent detection of diverse phage lifestyles within the same microbial communities [64].

This structural relationship is also evident at the level of host defense features. Although lysogens are enriched in complete CRISPR–Cas systems and higher spacer counts, virulent association does not significantly alter CRISPR prevalence and is accompanied only by a modest reduction in spacer abundance. Rather than supporting simple exclusion models in which adaptive immunity primarily limits prophage accumulation, these patterns suggest that defense signatures scale with prophage burden. Larger genomes may further contribute by providing expanded neutral integration sites and accessory capacity, thereby facilitating prophage retention [32]. Accordingly, virulent association is not driven by the absence of host defense systems, but may instead depend on the regulatory strength, activity state, or immune specificity of these systems [65, 66]. Thus, CRISPR prevalence appears more tightly linked to lysogen status than to virulent association alone.

Functional-trait analysis independently supports the host strategy axes identified above (Fig. 3). Across both the prophage-burden correlations and the comparison between virulent phage hosts and species without virulent phage associations, functional enrichments consistently converged on resource-acquisition modules, particularly substrate uptake and transport. Rather than emphasizing specific metabolic pathways, these enrichments favored acquisition of diverse small-molecules that can be directly imported into central metabolism, thereby reducing the likelihood that resource supply becomes limiting across heterogeneous environments [58, 59]. Functionally, these patterns corroborate the resource-acquisition potential captured by MCOA1 and suggest that the shared enrichment of prophage- and virulent-associated signals toward this strategy endpoint is more likely driven by long-term genomic allocation to resource input capacity.

### Limitations and future directions

Several potential limitations of our analysis merit attention. First, we did not include environmental metadata or community-level dynamics, so we cannot resolve how environmental factors and short-term ecological fluctuations influence prophage burden and virulent phage distributions.

We considered applying our framework to host genomes reconstructed from publicly available metagenomic datasets to evaluate environmental effects. However, many MAGs lacked sufficient completeness and quality for reliable estimation of the genomic traits analyzed here. In addition, environmental metadata associated with publicly available genomes were often incomplete, inconsistent, or difficult to standardize across datasets. We therefore adopted a conservative strategy and focused on high-quality reference genomes. This strategy improved the robustness of trait inference and reduced annotation artifacts, but it likely underrepresents environmentally derived bacterial diversity (e.g. there is a culturability bias because 99.69% of all genomes originated from cultivated isolates) and may limit the generality of our conclusions across the full spectrum of bacterial life histories.

A second limitation of our study is that our analyses rely on genome-based prophage identification, inference of virulent phage association, and functional trait annotation, each subject to method-specific biases and uncertainties. Inferences of virulent phage associations may be affected by incomplete representation of virulent phages in public databases, uncertainty and limited taxonomic resolution of host assignments derived from phage records, and the conservative lifestyle classification framework used in this study. Consequently, some virulent phage–host associations may have been missed. However, 88% of the host species identified by our framework were independently recovered by a stringent BacPHLIP/PhaTYP [67, 68] consensus dataset, and the same host-trait patterns were observed when the consensus dataset was projected onto the MCOA space. Thus, any resulting bias is likely to reflect conservative underestimation rather than inflation of virulent-phage associations. Consistent with this interpretation, species lacking detectable phage signals showed non-random distributions in host life-history strategy space (Fig. S7). We interpret these data as reflecting a combination of biological signal and methodological uncertainty rather than definitive evidence of phage-free bacterial lineages.

A third limitation of our study is that the dataset shows uneven phylogenetic representation, with certain phyla (e.g. *Pseudomonadota)* disproportionately represented. This reflects known biases in reference genome collections and cultivation efforts [55, 56], which could potentially influence large-scale patterns. Several aspects of our design mitigate these concerns. Key analyses defining the host life-history strategy space were performed at the GTDB species level by aggregating genomes within species, preventing overrepresentation of well-sampled species (e.g. multiple strains of *Escherichia coli*). Furthermore, stratified subsampling across both phylogenetic groups (phyla) and growth-strategy categories produced consistent MCOA structure, genome positioning, and phage-associated spatial patterns. Concordant gradients observed across analytical approaches also argue against a single methodological or database artifact driving the results.

Future improvements in metagenomic genome reconstruction, environmental metadata standardization, and phage lifestyle inference will enable broader representation of environmentally derived taxa and more complete characterization of phage–host associations. Integrating these advances with host-strategy-based frameworks will be necessary to better understand how host traits, environmental conditions, and phage lifestyles interact across ecosystems.

Overall, beyond extending existing ecological frameworks, our findings position host life-history strategy as an important complementary dimension in phage ecology. Host strategy composition may contribute to ecosystem-level variability in KtW- or PtW-like patterns, shaping long-term phage lifestyle patterns. Mapping bacterial isolates within this strategy space may guide the isolation of virulent phages for phage-based biocontrol. Because virulent enrichment is nested within prophage-rich backgrounds, hosts favorable for recovering virulent phages may also carry elevated prophage burdens and associated resistance or virulence genes. Incorporating host strategy information into phage isolation, therapeutic design, and ecosystem models may enhance predictive power and improve assessment of effectiveness in phage-based applications.

## Supplementary Material

supplementary_Information_6-24_final_submission_wrag168

## Data Availability

All data generated or analyzed during this study are included in this published article and its Supplementary Data files. Publicly available genome and phage datasets used in this study were obtained from NCBI, PhageScope, and GTDB. All scripts used for data processing and analysis are publicly available at https://github.com/ccw-rice/phage-host-trait-data.
