# Supplementary material for: Host life-history strategy is a critical determinant of virulent phage infection propensity
Source: ISME J. 2026 Jul 13;20(1):wrag168. doi: 10.1093/ismejo/wrag168 (PMC13374864; doi:10.1093/ismejo/wrag168)
Supplement: supplementary_Information_6-24_final_submission_wrag168 [file supplementary_information_6-24_final_submission_wrag168.docx]

**Supplementary Information**

**for**

**Host life-history strategy is a critical determinant of**

**virulent phage infection propensity**

Chuncheng Wu^a^, Jacques Mathieu^a^, Cory Schwarz^a^, Madelyn Whitaker^b^, Jenny A. Laverde Gomez^a^, Pedro J.J. Alvarez^a,b,c*^

^a^Department of Civil and Environmental Engineering, Rice University, Houston, Texas, USA

^b^Department of Chemical and Biomolecular Engineering, Rice University, Houston, Texas, USA

^c^ Rice WaTER Institute, Rice University, Houston, Texas, USA

*Address correspondence to Pedro J.J. Alvarez, alvarez@rice.edu

**Table of Contents**

**Supplementary Figures**

Figure S1. Key contributing variables to MCOA1…………………………………………3

Figure S2. Key contributing variables to MCOA2…………………………………………4

Figure S3. Prophage detection pipeline applied to 5,821 NCBI reference bacterial genomes

……………………………………………………………………………………………...5

Figure S4. Virulent phage identification pipeline applied to phage genomes from NCBI and PhageScope………………………………………………………………………………....6

Figure S5. Distribution of the number of prophages per genome………………………….7

Figure S6. Distribution of the average number and density of prophages per host genome as a function of bacterial genome size…………………………………………….…………….8

Figure S7. Species lacking detectable phage signals are enriched in host strategies quadrant characterized by slower growth and greater investment in cellular maintenance.…………9

Figure S8. Bootstrap-derived centroid positions of genomes by prophage count in MCOA space……………………………………………………………………………………….16

Figure S9. Spatial distribution of virulent phage host genomes across bootstrap iterations in MCOA space………………………………………………………………………………17

Figure S10. Prophage burden remains positively associated with resource-acquisition traits after removing predicted prophage regions from host genomes…………………………………18

**Supplementary Tables**

Table S1. Shared microTrait traits jointly associated with virulent phage presence and prophage accumulation…………………………………………………………………………………10

Table S2. Stability of variable contributions to MCOA axes across bootstrap iterations……12

Table S3. Stability of genome positions in MCOA space across bootstrap iterations………..14

**Supplementary Methods**

Assessment of robustness to taxonomic sampling bias……………………………………….11


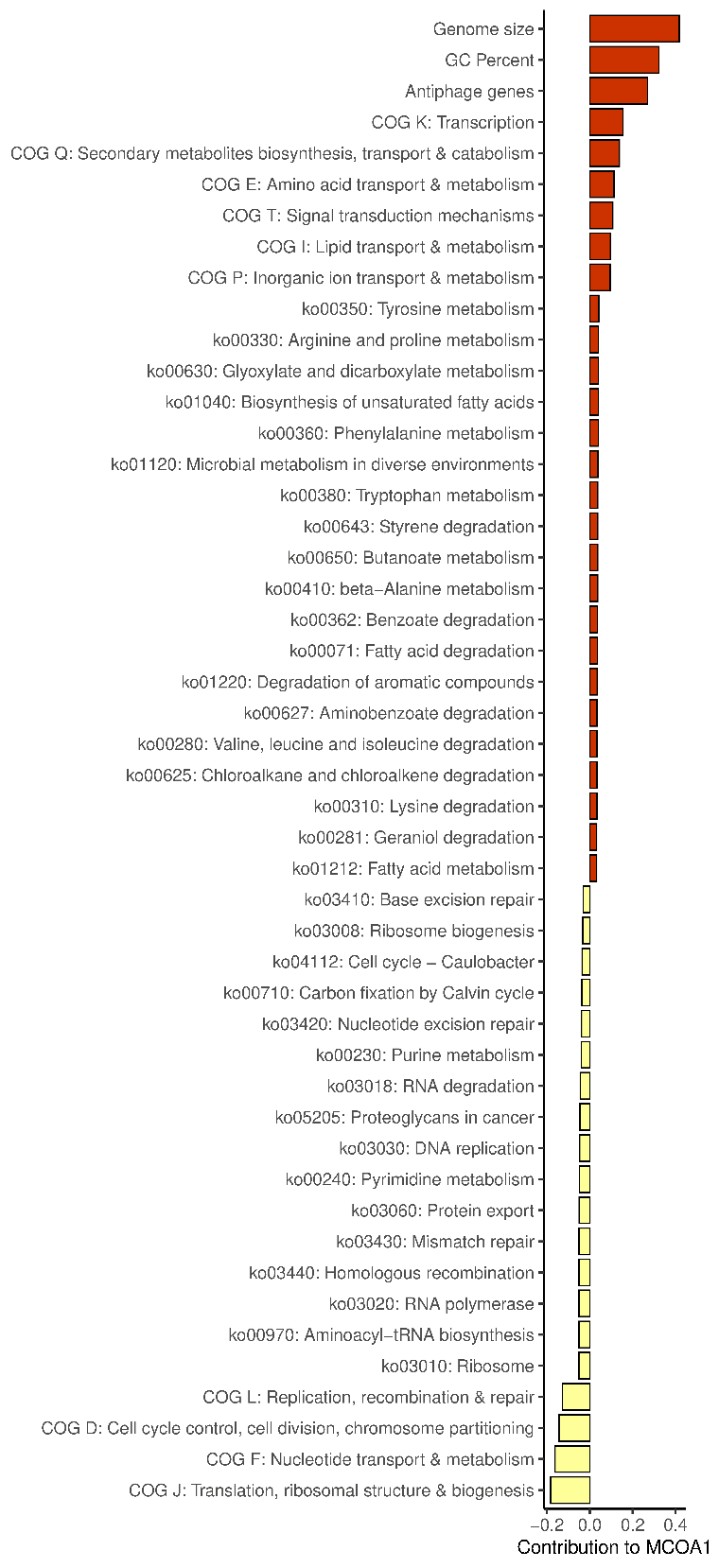


Figure S1. Key contributing variables to MCOA1. Positive contributions to MCOA1 were dominated by genome size and variables linked to metabolic expansion, including secondary metabolite biosynthesis, transport and catabolic functions, and amino acid and lipid metabolism. Negative contributions were primarily driven by information processing and core maintenance functions, including translation and ribosome biogenesis, replication and repair, and nucleotide-related processes.


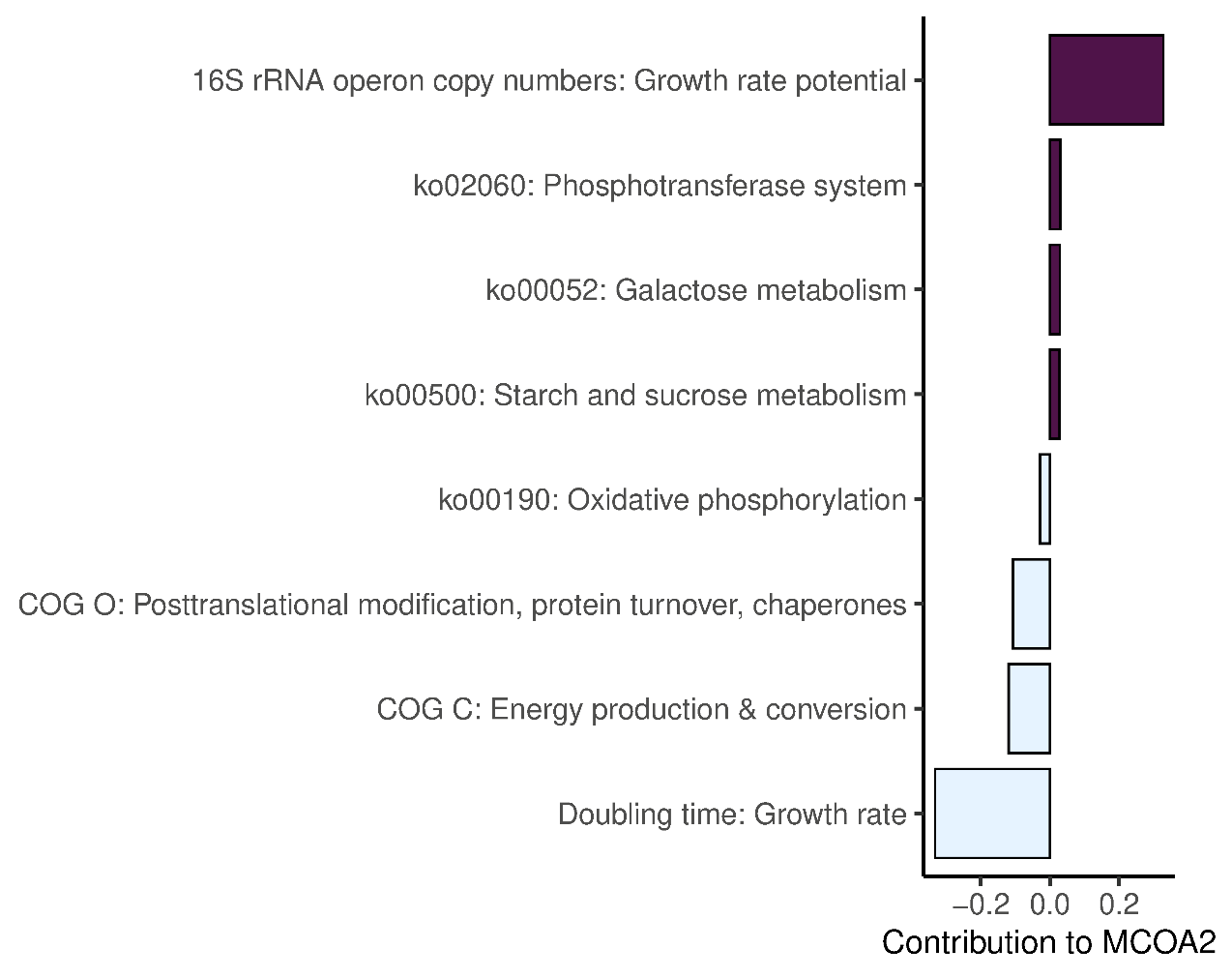


Figure S2. Key contributing variables to MCOA2. Generation time, 16S rRNA operon copy number, and representative metabolic pathways jointly defined MCOA2. Positive MCOA2 values were associated with higher growth potential and rapid uptake and metabolism of labile carbon sources (for example, phosphotransferase system and carbohydrate metabolism), whereas negative values were associated with energy production, post-translational modification and turnover, and cellular homeostasis maintenance.


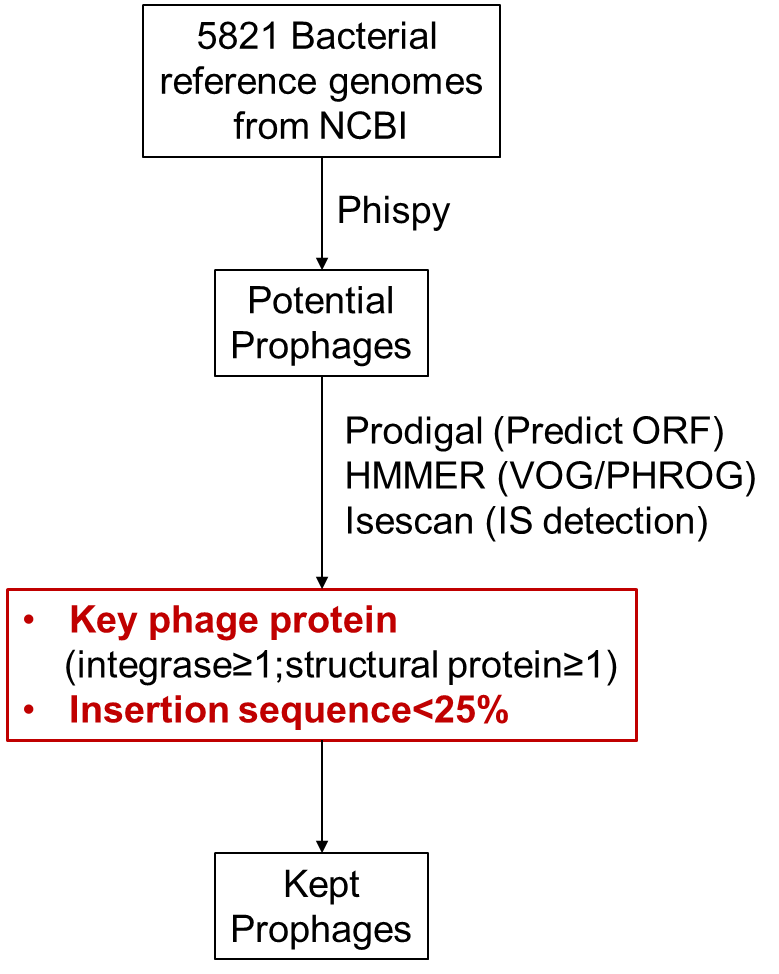
Figure S3. Prophage detection pipeline applied to 5,821 NCBI reference bacterial genomes.


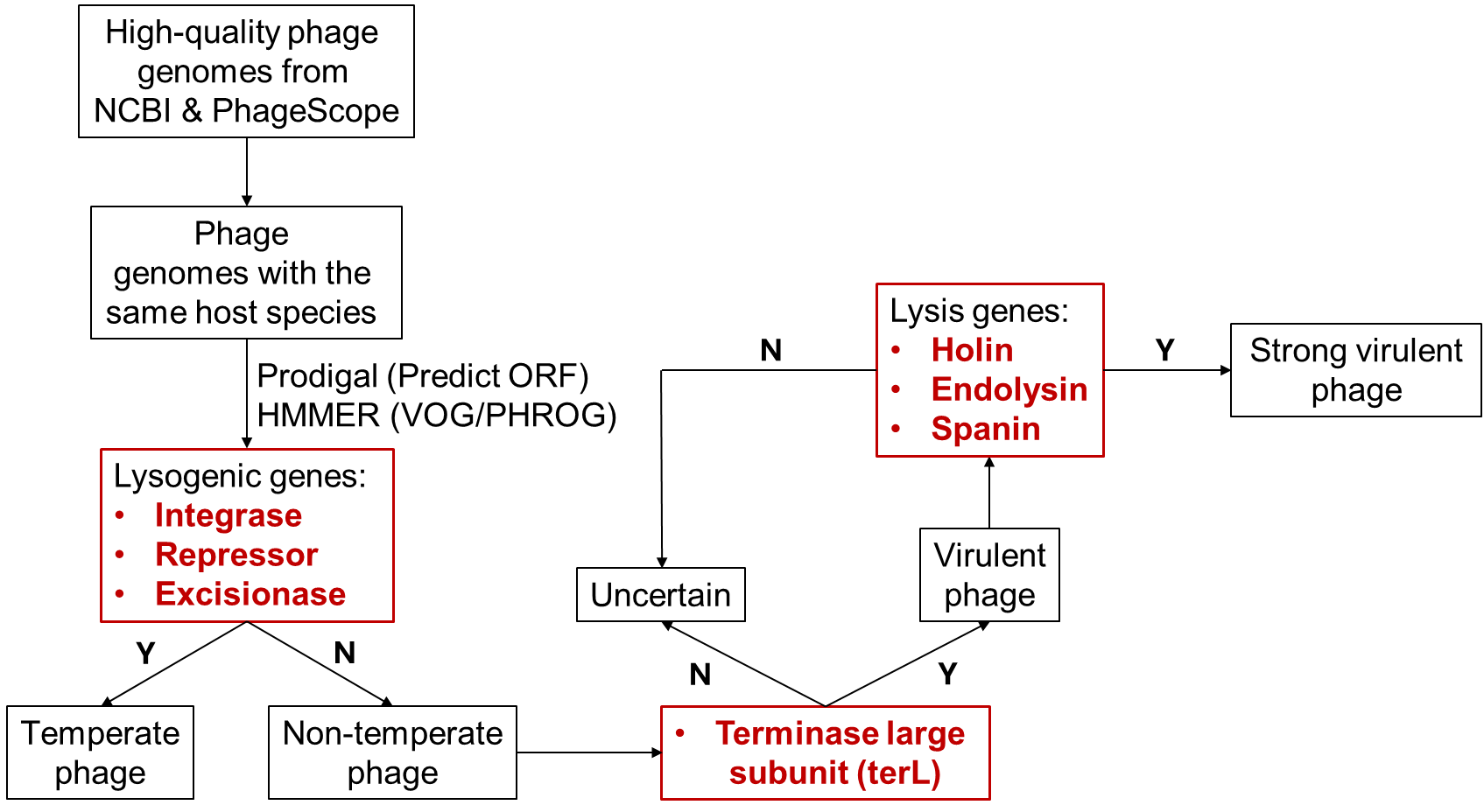


Figure S4. Virulent phage identification pipeline applied to phage genomes from NCBI and PhageScope.


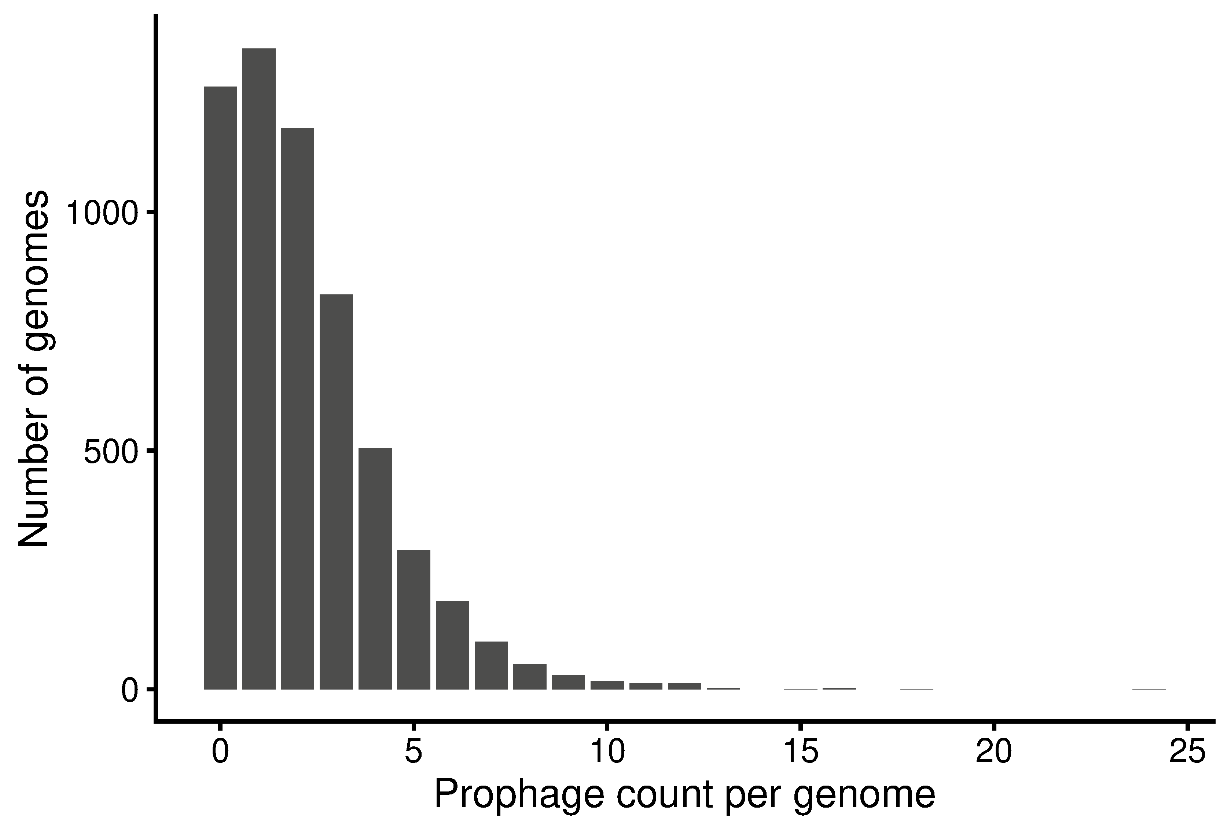


Figure S5. Distribution of the number of prophages per genome


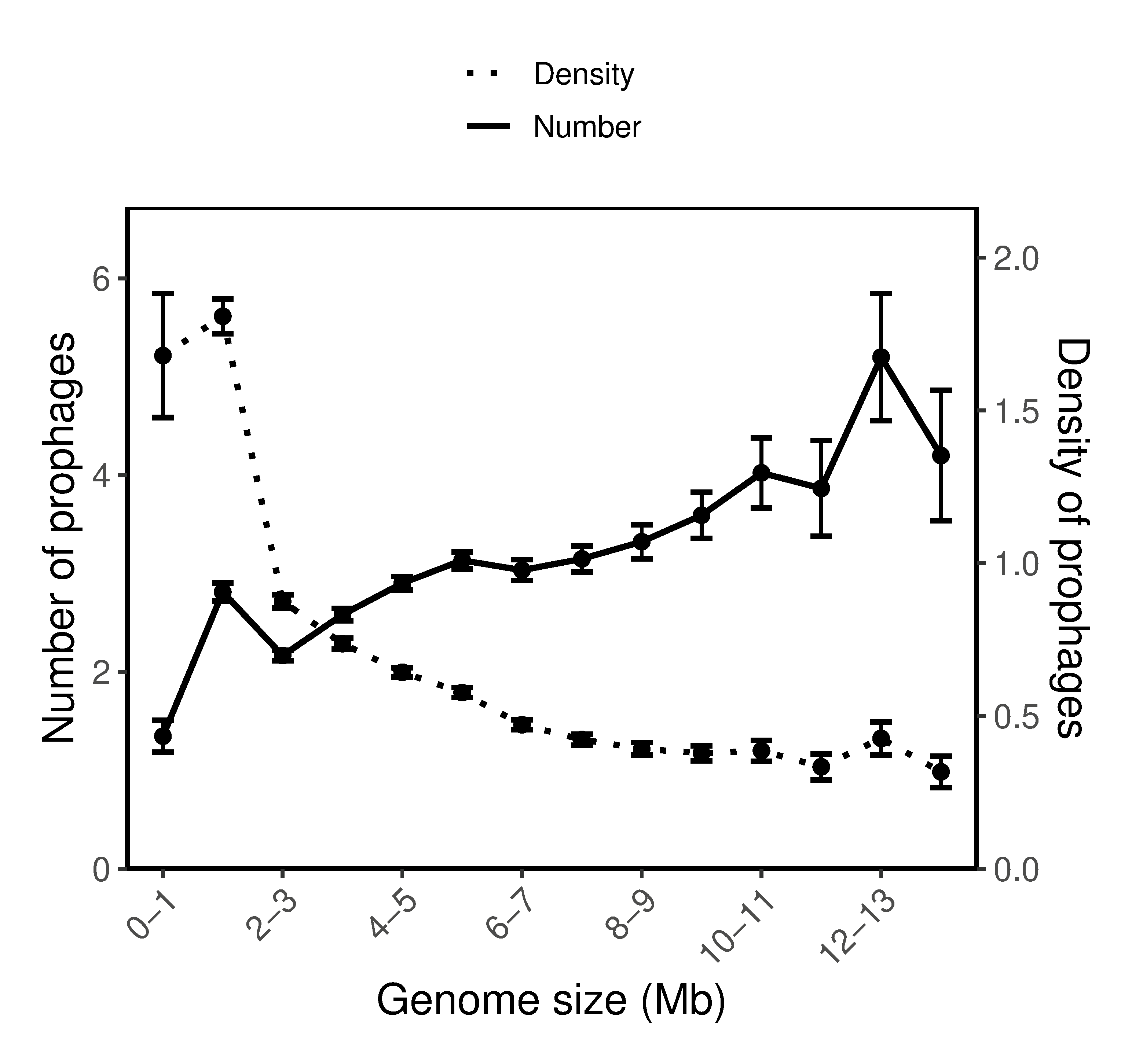


Figure S6. Distribution of the average number (full line) and density (dash line) of prophages per host genome in function of the size of the bacterial genome (Mb)


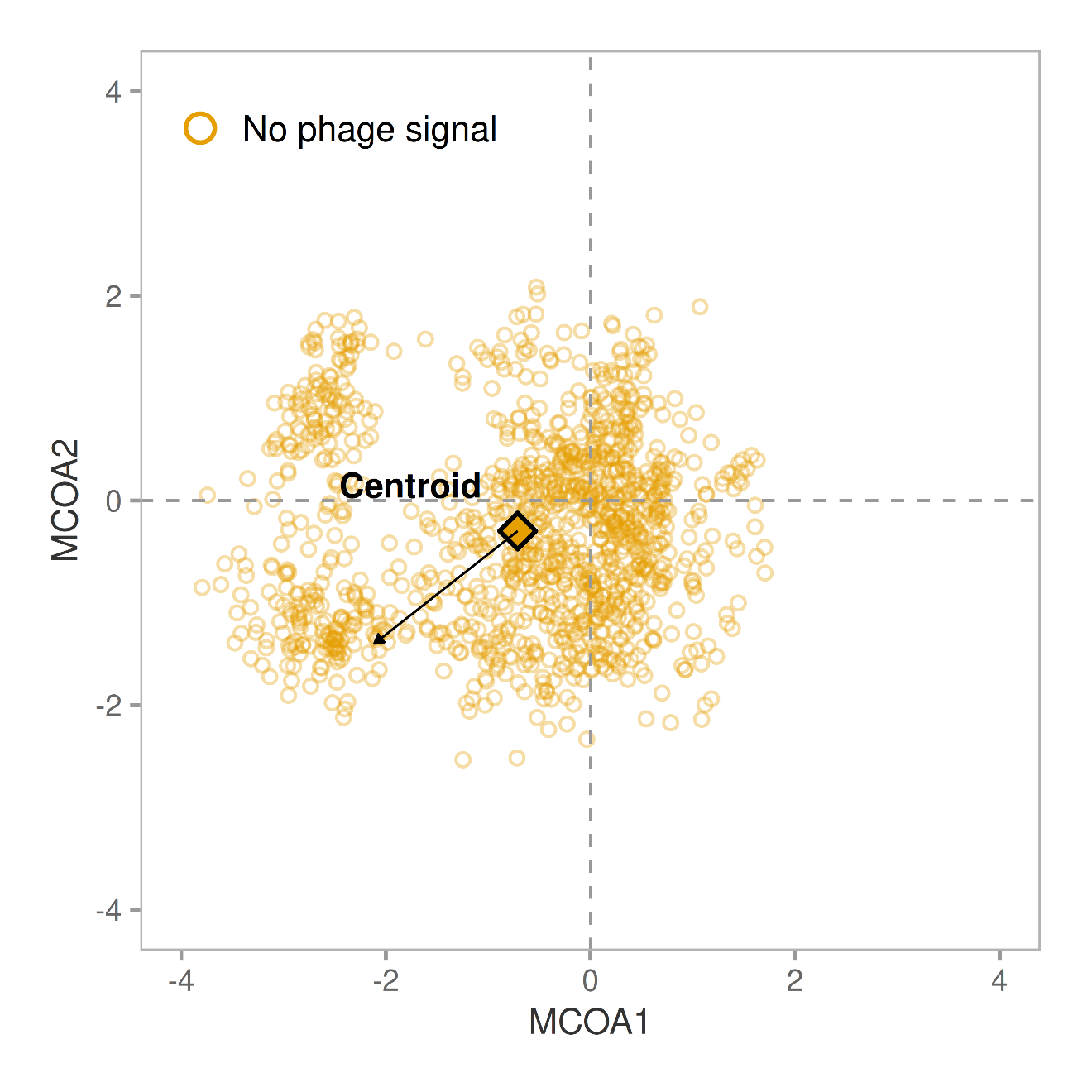


Figure S7. Species lacking detectable phage signals are enriched in host strategies quadrant characterized by slower growth and greater investment in cellular maintenance.

**Table S1 Shared microTrait traits jointly associated with virulent phage presence and prophage accumulation.**

| **microTrait count trait** | **Selection criteria** |
| --- | --- |
| Resource Acquisition: monosaccharide transport | Enriched in virulent phage hosts; positively correlated with prophage burden  (Retained if q ≤ 0.01 in both tests, \|Cliff’s delta\| ≥ 0.20, and Spearman’s ρ ≥ 0.20.) |
| Resource Acquisition: vitamin B transport |  |
| Resource Acquisition: free amino acids transport |  |
| Resource Acquisition: nucleobase transport |  |
| Resource Acquisition: dicarboxylate transport |  |
| Resource Acquisition: monocarboxylate transport |  |
| Resource Acquisition: oligosaccharide transport |  |
| Resource Acquisition: sugar alcohol transport |  |
| Resource Acquisition: amino sugar transport |  |
| Resource Acquisition: peptide transport |  |
| Resource Acquisition: carbohydrate acid transport |  |

**Supplementary Methods: Assessment of robustness to taxonomic sampling bias**

To assess the robustness of the MCOA-derived host life-history strategy space and associated phage patterns to taxonomic bias and uneven representation of host growth strategies, a stratified bootstrap subsampling analysis was performed. In each iteration, species were first stratified into fast-growing (predicted doubling time < 5 h) and slow-growing (≥ 5 h) groups, from which 500 species were sampled from each group using a phylum-balanced procedure. MCOA was then recomputed for each dataset. The stability of MCOA space, genome positions, and downstream spatial patterns was evaluated across iterations.

A total of 50 bootstrap iterations were performed. For each resampled dataset, the full MCOA analysis and downstream workflow were repeated. We evaluated robustness at three levels:

1. Stability of MCOA axes and contributing variables

The consistency of major contributing variables to MCOA1 and MCOA2 was assessed across bootstrap runs by tracking variable identity and ranking.

1. Stability of genome positions in MCOA space

Genome coordinates from bootstrap runs were matched to those in the full dataset based on genome identifier. Spearman correlations were computed for MCOA1 and MCOA2 coordinates. In addition, Euclidean displacement was calculated for each genome.

1. Stability of phage-associated spatial patterns

For prophage burden, genomes were grouped by prophage count, and centroid trajectories were computed across bootstrap runs to evaluate the persistence of the continuous gradient in MCOA space. For virulent phage-associated genomes, centroid positions were calculated for genomes with virulent phage signals (virulent count > 0) across bootstrap runs to assess spatial consistency.

All analyses were conducted in R using custom scripts. Summary statistics across bootstrap iterations, including axis correlations, centroid positions, and trajectory variability, were shown below and used to evaluate robustness.

1. Stability of MCOA axes and contributing variables

Across all bootstrap iterations, the major contributors to MCOA axes remained highly consistent. The top 63% of variables contributing to MCOA1 showed no substantial changes in identity or ranking, while the top 20% of variables for MCOA2 were preserved. These results indicate that the primary ecological gradients captured by MCOA are not driven by taxonomic overrepresentation.

**Table S2. Stability of variable contributions to MCOA axes across bootstrap iterations**

| Variable | Axis | Frequency across 50 runs | Mean rank among top contributors | Whether in original dataset |
| --- | --- | --- | --- | --- |
| total_sequence_length | 1 | 1.00 | 1.00 | TRUE |
| gc_percent | 1 | 1.00 | 2.00 | TRUE |
| sum_of_antiphage_genes | 1 | 1.00 | 3.00 | TRUE |
| j | 1 | 1.00 | 4.00 | TRUE |
| f | 1 | 1.00 | 5.00 | TRUE |
| k | 1 | 1.00 | 6.00 | TRUE |
| q | 1 | 1.00 | 7.40 | TRUE |
| d | 1 | 1.00 | 7.60 | TRUE |
| s | 1 | 1.00 | 9.00 | TRUE |
| ko03010 | 1 | 1.00 | 11.02 | TRUE |
| ko00970 | 1 | 1.00 | 12.44 | TRUE |
| ko03060 | 1 | 1.00 | 12.60 | TRUE |
| ko03440 | 1 | 1.00 | 14.38 | TRUE |
| ko03430 | 1 | 1.00 | 15.26 | TRUE |
| ko03030 | 1 | 1.00 | 15.74 | TRUE |
| ko00240 | 1 | 1.00 | 18.02 | TRUE |
| ko03020 | 1 | 1.00 | 18.78 | TRUE |
| ko00350 | 1 | 1.00 | 20.42 | TRUE |
| ko04112 | 1 | 1.00 | 22.22 | TRUE |
| ko00330 | 1 | 1.00 | 22.48 | TRUE |
| ko01040 | 1 | 1.00 | 23.96 | TRUE |
| ko00630 | 1 | 1.00 | 26.08 | TRUE |
| ko03420 | 1 | 1.00 | 26.24 | TRUE |
| ko00360 | 1 | 1.00 | 26.72 | TRUE |
| ko00908 | 1 | 1.00 | 27.56 | TRUE |
| ko05205 | 1 | 1.00 | 27.94 | TRUE |
| ko01120 | 1 | 1.00 | 28.94 | TRUE |
| ko03018 | 1 | 1.00 | 31.06 | TRUE |
| ko01220 | 1 | 1.00 | 31.54 | TRUE |
| ko00643 | 1 | 1.00 | 33.76 | TRUE |
| ko00410 | 1 | 1.00 | 33.96 | TRUE |
| ko00625 | 1 | 1.00 | 34.38 | TRUE |
| ko00380 | 1 | 1.00 | 35.68 | TRUE |
| ko00340 | 1 | 1.00 | 37.92 | FALSE |
| ko00362 | 1 | 1.00 | 38.40 | TRUE |
| ko00982 | 1 | 1.00 | 38.82 | FALSE |
| ko00830 | 1 | 1.00 | 40.60 | FALSE |
| ko00071 | 1 | 1.00 | 40.70 | TRUE |
| ko00280 | 1 | 1.00 | 41.68 | TRUE |
| ko01212 | 1 | 0.98 | 45.27 | TRUE |
| ko00626 | 1 | 0.96 | 43.38 | FALSE |
| ko00981 | 1 | 0.90 | 45.40 | FALSE |
| ko00230 | 1 | 0.82 | 44.37 | TRUE |
| ko00364 | 1 | 0.80 | 44.83 | FALSE |
| ko00333 | 1 | 0.78 | 46.23 | FALSE |
| ko00980 | 1 | 0.74 | 47.38 | FALSE |
| ce11 | 1 | 0.64 | 17.59 | FALSE |
| ko00260 | 1 | 0.62 | 46.23 | FALSE |
| l | 1 | 0.54 | 10.11 | TRUE |
| gt19 | 1 | 0.52 | 19.50 | FALSE |
| gt28 | 1 | 0.50 | 15.28 | FALSE |
| ko01502 | 1 | 0.40 | 47.35 | FALSE |
| ko01130 | 1 | 0.32 | 46.56 | FALSE |
| gt1 | 1 | 0.26 | 20.08 | FALSE |
| i | 1 | 0.24 | 10.50 | TRUE |
| ko00910 | 1 | 0.22 | 48.55 | FALSE |
| ko00550 | 1 | 0.20 | 45.30 | FALSE |
| ko00650 | 1 | 0.16 | 48.38 | TRUE |
| ko00281 | 1 | 0.16 | 48.63 | TRUE |
| ko00623 | 1 | 0.08 | 49.00 | FALSE |
| ko00361 | 1 | 0.04 | 49.00 | FALSE |
| t | 1 | 0.02 | 11.00 | TRUE |
| gt30 | 1 | 0.02 | 20.00 | FALSE |
| ko00592 | 1 | 0.02 | 49.00 | FALSE |
| ko00642 | 1 | 0.02 | 50.00 | FALSE |
| ko03008 | 1 | 0.02 | 50.00 | TRUE |
| ko00220 | 1 | 0.02 | 50.00 | FALSE |
| 16s_rrna_count | 2 | 1.00 | 1.34 | TRUE |
| doubling_time_hour | 2 | 1.00 | 1.66 | TRUE |
| c | 2 | 0.26 | 3.00 | TRUE |
| ko00052 | 2 | 0.02 | 4.00 | TRUE |

* Frequency across 50 runs indicates the proportion of bootstrap runs (n = 50) in which each variable was identified among the top contributors. Mean rank represents the average rank of the variable across runs. “Whether in original dataset” indicates whether the variable was also identified as a top contributor in the full dataset.

1. Stability of genome positions in MCOA space

Genome positions in the reduced MCOA space were also highly stable across resampled datasets. Despite reduced sample size in each bootstrap iteration, individual genomes retained their relative positions within the ordination space, indicating that the global structure of the host life-history landscape is robust to subsampling.

**Table S3. Stability of genome positions in MCOA space across bootstrap iterations**

| Bootstrap_run | MCOA1_correlation | MCOA2_correlation | Mean_displacement |
| --- | --- | --- | --- |
| run_01 | 0.99 | 0.98 | 0.66 |
| run_02 | 0.99 | 0.97 | 0.66 |
| run_03 | 0.99 | 0.98 | 0.65 |
| run_04 | 0.99 | 0.98 | 0.67 |
| run_05 | 0.99 | 0.98 | 0.67 |
| run_06 | 0.99 | 0.98 | 0.67 |
| run_07 | 0.99 | 0.97 | 0.67 |
| run_08 | 0.99 | 0.99 | 0.62 |
| run_09 | 0.99 | 0.99 | 0.65 |
| run_10 | 0.99 | 0.98 | 0.67 |
| run_11 | 0.99 | 0.98 | 0.66 |
| run_12 | 0.99 | 0.98 | 0.68 |
| run_13 | 0.99 | 0.98 | 0.66 |
| run_14 | 0.99 | 0.99 | 0.65 |
| run_15 | 0.99 | 0.98 | 0.66 |
| run_16 | 1.00 | 0.98 | 0.65 |
| run_17 | 0.99 | 0.98 | 0.68 |
| run_18 | 0.99 | 0.98 | 0.67 |
| run_19 | 0.99 | 0.98 | 0.68 |
| run_20 | 0.99 | 0.98 | 0.65 |
| run_21 | 0.99 | 0.99 | 0.65 |
| run_22 | 0.99 | 0.98 | 0.65 |
| run_23 | 0.99 | 0.97 | 0.67 |
| run_24 | 0.99 | 0.99 | 0.65 |
| run_25 | 0.99 | 0.98 | 0.65 |
| run_26 | 0.99 | 0.98 | 0.67 |
| run_27 | 0.99 | 0.97 | 0.65 |
| run_28 | 0.99 | 0.98 | 0.65 |
| run_29 | 0.99 | 0.98 | 0.67 |
| run_30 | 0.99 | 0.98 | 0.66 |
| run_31 | 0.99 | 0.99 | 0.65 |
| run_32 | 0.99 | 0.98 | 0.66 |
| run_33 | 0.99 | 0.99 | 0.66 |
| run_34 | 0.99 | 0.98 | 0.65 |
| run_35 | 0.99 | 0.96 | 0.68 |
| run_36 | 0.99 | 0.98 | 0.66 |
| run_37 | 0.99 | 0.97 | 0.66 |
| run_38 | 0.99 | 0.98 | 0.68 |
| run_39 | 0.99 | 0.99 | 0.65 |
| run_40 | 0.99 | 0.98 | 0.66 |
| run_41 | 0.99 | 0.98 | 0.65 |
| run_42 | 0.99 | 0.98 | 0.67 |
| run_43 | 0.99 | 0.98 | 0.65 |
| run_44 | 0.99 | 0.98 | 0.66 |
| run_45 | 0.99 | 0.98 | 0.69 |
| run_46 | 0.99 | 0.98 | 0.66 |
| run_47 | 0.99 | 0.98 | 0.64 |
| run_48 | 0.99 | 0.98 | 0.66 |
| run_49 | 0.99 | 0.98 | 0.66 |
| run_50 | 0.99 | 0.98 | 0.63 |

*For each bootstrap run, Spearman correlations (MCOA1 and MCOA2) quantify the consistency of genome positions along each axis relative to the full dataset. Mean displacement represents the average Euclidean distance between genome positions in bootstrap and full datasets.

1. Stability of phage-associated spatial patterns

**
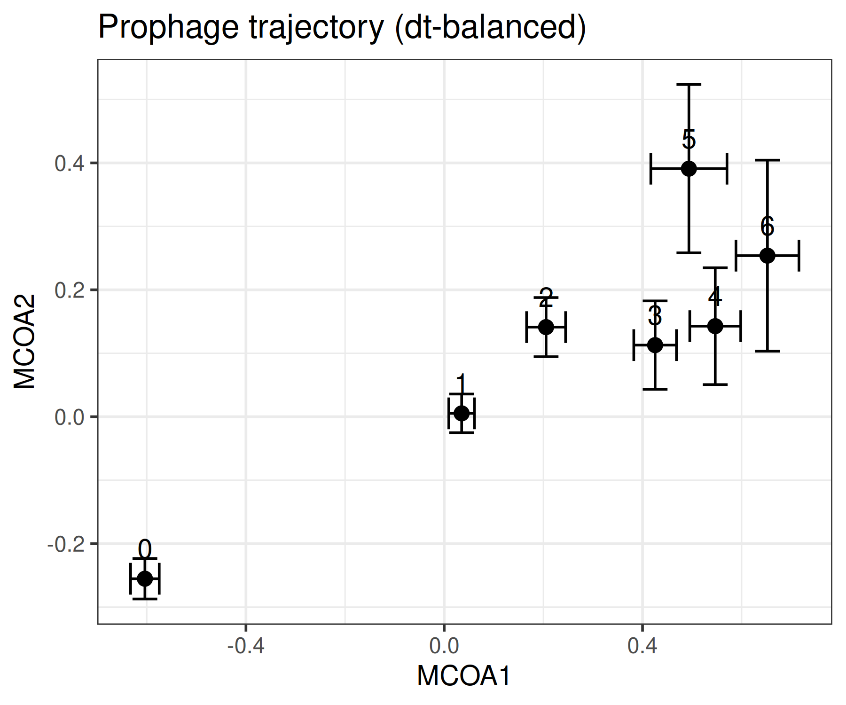
**The continuous displacement of prophage-count centroids across MCOA space was consistently reproduced across all bootstrap runs (Figure S8). Genomes with increasing prophage counts shifted along a similar trajectory from the lower-left to the upper-right region of the MCOA space, consistent with the pattern observed in the full dataset. While reduced sample size led to increased variability along MCOA2, likely due to diminished contributions of variables (20% preserved), the primary gradient along MCOA1 remained stable.

**Figure S8. Bootstrap-derived centroid positions of genomes by prophage count in MCOA space.** Each point represents the mean position of genomes within a given prophage count category, calculated across 50 bootstrap subsamples. Numbers indicate the prophage count group. Error bars represent the standard deviation of centroid positions across bootstrap iterations along each axis.

Genomes of virulent phage hosts were relatively rare in the dataset and therefore further reduced under subsampling. Despite this, their spatial distribution remained broadly consistent across bootstrap runs (Figure S9). Although minor shifts were observed, primarily as a leftward displacement along MCOA1, the overall location of virulent phage host genomes within the host life-history space was preserved, indicating that their ecological positioning is not driven by the overrepresentation of specific taxa.

**
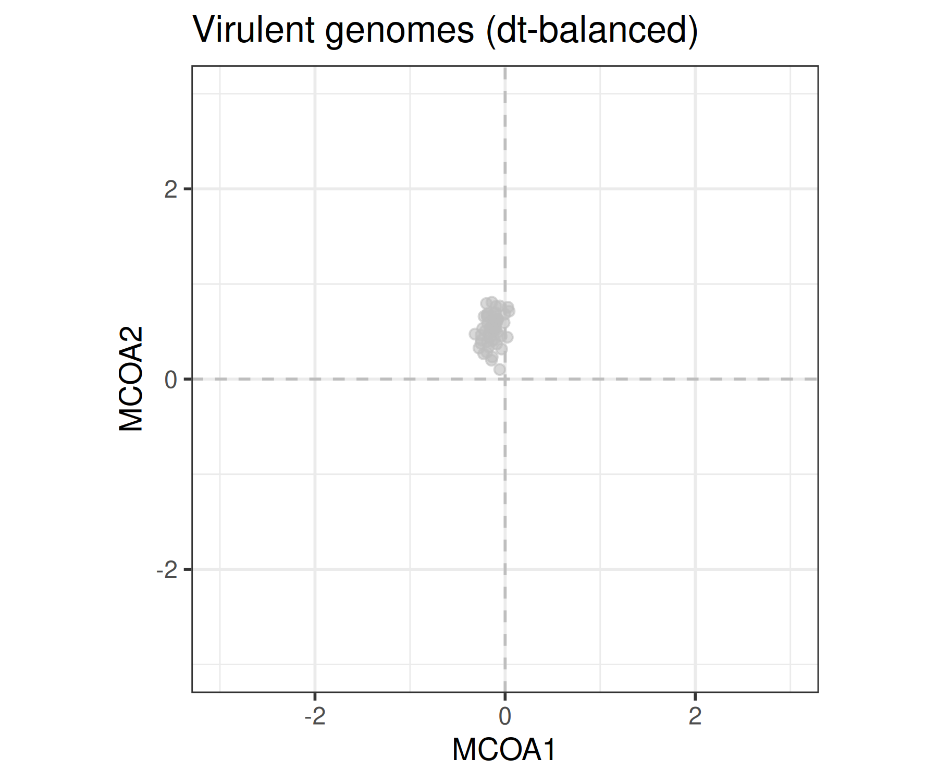
**

**Figure S9. Spatial distribution of virulent phage host genomes across bootstrap iterations in MCOA space.** Each point represents the centroid of genome associated with virulent phages (virulent count > 0) in a single bootstrap subsample.


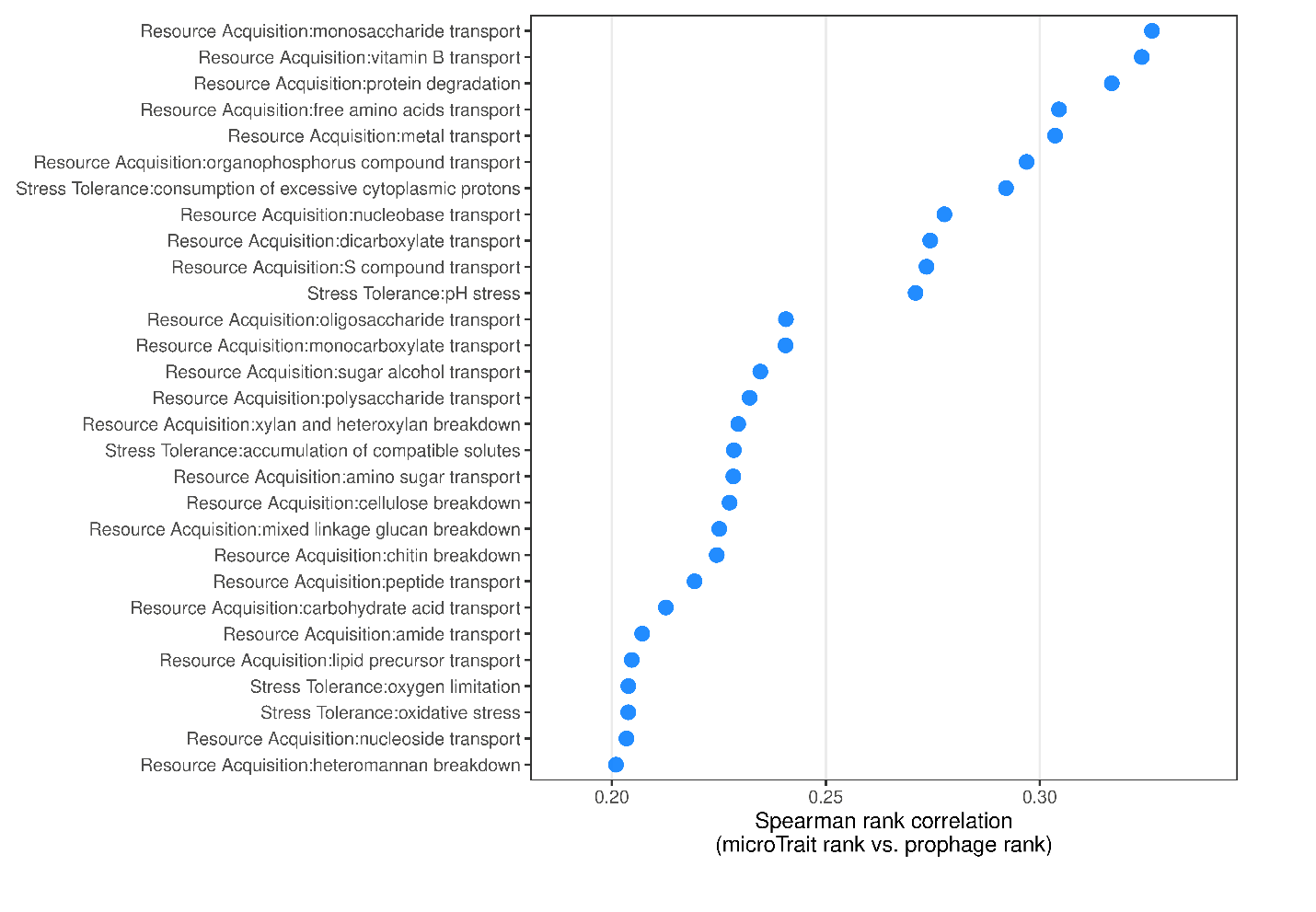


Figure S10. Prophage burden remains positively associated with resource-acquisition traits after removing predicted prophage regions from host genomes. Monotonic associations between microTrait traits and prophage burden were quantified using Spearman’s rank correlation (ρ) at the GTDB species level, followed by false discovery rate (FDR) correction. Each point represents one microTrait category, with the x axis indicating Spearman’s ρ between microTrait rank and prophage rank. Traits with q ≤ 0.01 and |ρ| ≥ 0.20 were retained and visualized.
